# Molecular Motions in Functional Self-Assembled Nanostructures

**DOI:** 10.3390/ijms14022303

**Published:** 2013-01-24

**Authors:** Alexandre Dhotel, Ziguang Chen, Laurent Delbreilh, Boulos Youssef, Jean-Marc Saiter, Li Tan

**Affiliations:** 1 AMME–LECAP, EA4528, International Laboratory, Institut des Matériaux de Rouen, Université et INSA de Rouen, BP12, 76801 Saint Etienne du Rouvray Cedex, France; E-Mails: alexandredhotel@gmail.com (A.D.); laurent.delbreilh@univ-rouen.fr (L.D.); boulos.youssef@univ-rouen.fr (B.Y.); jean-marc.saiter@univ-rouen.fr (J.-M.S.); 2 AMME–A-TEAM, Department of Mechanical and Materials Engineering, University of Nebraska, Lincoln, NE 68588, USA; E-Mail: chenziguang@huskers.unl.edu

**Keywords:** self-assembly, nanostructures, molecular motion, molecular recognition, stimuli-responsive materials, metal-organic frameworks (MOFs), DNA, self-assembled monolayers (SAMs), molecular rotors, block copolymers

## Abstract

The construction of “smart” materials able to perform specific functions at the molecular scale through the application of various stimuli is highly attractive but still challenging. The most recent applications indicate that the outstanding flexibility of self-assembled architectures can be employed as a powerful tool for the development of innovative molecular devices, functional surfaces and smart nanomaterials. Structural flexibility of these materials is known to be conferred by weak intermolecular forces involved in self-assembly strategies. However, some fundamental mechanisms responsible for conformational lability remain unexplored. Furthermore, the role played by stronger bonds, such as coordination, ionic and covalent bonding, is sometimes neglected while they can be employed readily to produce mechanically robust but also chemically reversible structures. In this review, recent applications of structural flexibility and molecular motions in self-assembled nanostructures are discussed. Special focus is given to advanced materials exhibiting significant performance changes after an external stimulus is applied, such as light exposure, pH variation, heat treatment or electromagnetic field. The crucial role played by strong intra- and weak intermolecular interactions on structural lability and responsiveness is highlighted.

## 1. Introduction

Molecular self-assembly (MSA) offers a molecular-level control of material structure, composition, morphology and dimensions, thus enabling the construction of an extensive variety of 1-, 2- and 3-D nanostructures [1–4]. Astonishing efforts have been carried out to elaborate processes able to fabricate organized materials with nanometer resolution, which found applications in protective coatings [5], molecular electronic devices [6,7], sensors [8,9], robust textiles [10], and biofunctional materials [11].

Besides these powerful “passive” applications, dynamic and environment-sensitive properties of self-assembled architectures are continuously gaining interest. Non-covalent physical interactions that drive the assembly of building blocks can be readily dissociated and re-associated, thus conferring outstanding flexibility to the final supramolecular structure. Recent advances indicate that taking advantage of the specificity, geometry, strength and sometimes, the reversibility of these interactions enables the elaboration of highly functional and stimuli-sensitive materials. More interestingly, stronger chemical interactions, which are often referred to as irreversible bonds, can also exhibit a certain degree of lability under specific conditions thus allowing the formation of both robust and dynamic materials. Molecular motions and bond lability are therefore now regarded as powerful tools for the construction of “active” materials. However, a better understanding of mechanisms inducing flexibility is required to enlarge potential applications in a near future.

In the present review, we aim to compile recent advances in the use of molecular motions to functionalize self-assembled nanostructures. Dynamic properties are discussed according to material nature, structure and bond flexibility. Relevant examples are summarized and classified into three categories of widely investigated self-assembled materials: (i) fully organic; (ii) organic-inorganic hybrid; (iii) metal-organic structures. Special emphasis is given to the crucial role played by strong and weak bonding on structural flexibility and responsiveness of the final product.

## 2. Flexibility of Bonding in Self-Assembled Systems

Non-covalent intermolecular bonds are known to govern self-assembly of molecules through a balance of attractive and repulsive interactions leading to the formation of larger and ordered supramolecules. Understanding the fundamental principles responsible for molecular self-assembly is still challenging due to numerous, complex and specific interplays between multiple subunits or building blocks. In order to engineer dynamic or reconfigurable systems, the key role of some weak non-covalent bonds involved in self-assembly processes have received continued attentions and is now well-documented [12–15]. Such interactions not only rule the assembly process leading to the formation of the ordered architecture but also control the dynamic behavior and properties of the final supramolecule. In this section, physical and chemical bonds commonly encountered in self-assembled systems are briefly described. A special focus is given to the lability, conformational flexibility and sometimes reversibility of supramolecular architectures.

### 2.1. Weak Interactions

Weak interactions, such as van der Waals forces, hydrogen bonds and π interactions are among the most prevalent low-energy forces encountered in self-assembled systems. Their weakness and reversibility play a crucial role in the self-assembly process of single or multiple types of building blocks. Generation of ordered structures through these interactions requires the building blocks to move and adjust their initial position even after aggregation. This adjustability is necessary for the final system to reach its lowest-energy state and achieve a nearly perfect organized structure. Essentially, weak bonds holding components in a self-assembled structure are the reasons for structural flexibility [16].

Among the weak interactions, van der Waals (vdW) forces are the weakest and most ubiquitous interactions at the nanoscale. These forces can be classified into several types depending on the nature of components but are generally caused by the competition between repulsive and attractive interactions between two components with random fluctuations of electric charges. Such fluctuations induce the polarization of the electron shells thus making individual component temporarily polar and causing an opposite polarization in its neighboring body. Oppositely polarized components are then attracted toward each other until, according to the Pauli exclusion principle, they are strongly repulsed due to overlapping of orbitals. Typical energies of such electrostatic interactions vary between <1 and 4 kJ·mol^−1^ making their dissociation plausible and easy. Despite their trivial strength, such ever-present forces have a significant influence on the ordering and cohesion of alkyl chains within self-assembled monolayers [17,18] and multilayered films [19–22].

Stronger than van der Waals forces, hydrogen bonding is a special type of dipole-dipole interaction. Even though globally neutral, molecules can have electric dipoles bearing a distribution of partial positive and negative charges. Interactions between these dipoles guide molecules to orient so that attractive interactions are maximized and repulsive interactions minimized. Hydrogen bonds are formed when a hydrogen atom covalently bonded to an electronegative atom (hydrogen donor or electron acceptor) interacts with the lone pair of electrons from another electronegative atom (hydrogen acceptor or electron donor), which comes either from an adjacent molecule or within the former molecule itself. The strength of the hydrogen bond being dependent on both the donor-acceptor distance and their electronegativities, a typical bond energy varies between 4 and 30 kJ·mol^−1^. Even though mildly strong, hydrogen bonds are directional and can greatly affect the physicochemical properties as well as the ordering of building blocks within self-assembled systems [23–25] and solid crystals [26]. The reversibility and weakness of these bonds is also widely employed for the design and synthesis of self-healing materials [27] and the fabrication of pH-erasable thin films for drug delivery and biosensors [28]. In biological systems, hydrogen bonding also plays a central role in the folding and stabilization of proteins [29,30] as well as in the specific interactions between DNA strands [31–33].

Another kind of weak interaction frequently found in the literature is hydrophobic interaction, also called hydrophobic bonding. Even though this type of interaction causes the apparent repulsion between water and hydrocarbon molecules, it cannot be directly related to the formation of physical bonds. The hydrophobic effect, which is one of the major driving forces for the formation of lipid bilayers and micelles, results from the contributions of both van der Waals forces between hydrocarbon compounds and hydrogen bonding between water molecules [34,35]. Therefore, the term “hydrophobic effect” should be preferred to “hydrophobic bonding”.

A strong correlation can easily be made between hydrogen and halogen bonding as they both result from the interaction between electron donor and electron acceptor. In a halogen bond, an electrophilic region on a halogen atom (electron acceptor) interacts with a nucleophilic region of a molecule, or molecular fragment (electron donor). While rarely exploited in the past to direct the formation of supramolecular assemblies, halogen bonding is becoming increasingly important in supramolecular chemistry because of its directionality and tuneable strength [36].

**Figure 1:**
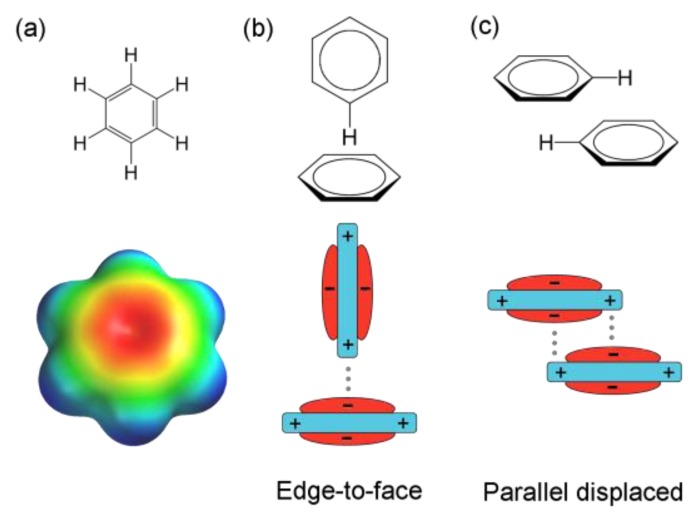
(**a**) Electrostatic potential surface of a benzene molecule (Spartan, B3LYP/6-31G*) (**b**) and (**c**) schematic representations of interaction geometries of a benzene dimer.

Another type of weak interactions commonly observed in self-assembled systems is π interaction. This general term refers to the non-covalent interaction involving a π-electron-rich unit with a cation, an anion, or another π-system. The latter, namely π–π interaction or π stacking, is defined as an attractive interaction between two stacked aromatic rings. Such interactions are caused by basic electrostatic interactions as described by the widely accepted model established by Hunter and Sanders in 1990 [37]. Electrons in π bonds of aromatic rings form a quadrupole moment (*i.e.*, two dipoles aligned so that no net dipole can be distinguished) due to the stronger electronegativity of *sp*^2^ carbons compared to hydrogen atoms [38]. In the case of benzene, this quadrupole creates a partial negative charge on both faces of the π system and a partial positive charge around the aromatic ring (Figure 1a). According to this description, a face-centered stacking of π systems on top of each other would be energetically unfavorable and therefore not stable. However, aromatic rings can interact with each other through the edge-to-face (also known as T-shaped or edge-on) geometry or the parallel displaced (also referred to as parallel off-centered) stacking (Figure 1b,c) where regions of negative electrostatic potential (faces) can interact with regions of positive electrostatic charges (periphery) [37–41]. The face-centered stacking geometry suggested by the terms “π-stacking” and “π–π interactions” being misleading, the use of these terms is now questioned [40,42]. Depending on the chemical nature of units, interactions between π-systems have typical energies between 5 and 40 kJ·mol^−1^. In self-assembled systems, interactions between π-units are often used in organic electronics [43] and to promote and stabilize molecular packing [22,44,45]. Their reversibility and flexibility are also commonly employed in host-guest assemblies [39,46] and recently, for the specific attachment of carbon nanotubes on surface [47]. In biological systems, interactions between aromatic rings are largely involved in protein–ligand complexation [48] and stabilization of the double helical structure of DNA [31,49].

### 2.2. Strong Bonding

Strong bonds, also known as intramolecular forces or chemical bonding, are several orders of magnitude stronger than weak interactions described above. Despite their strength, strong bonding such as ionic, covalent and coordination bonds can play a central role in the formation of self-assembled materials and also contribute tremendously on the dynamic properties of the final supramolecule.

Ionic bonding, also referred to as electrostatic interactions between two oppositely charged ions, leads to the complete transfer of one or more valence electrons from the negatively charged anion to the positively charged cation in order for them to reach a stable electronic configuration. The strength of interaction between ions is directly related to their charges and can be determined by measuring the lattice energy of the compound. Typical binding energies vary between several hundreds to thousands kilojoules per mole ensuring the cohesion within the ionic structure. Even though remarkably strong in solid state, interactions between ions are not directional and can be readily interrupted or broken in a wet environment, for instance, water dissolution of ionic solids. This versatility makes ionic bonding of great interest for the conception of mechanically robust but chemically reversible supramolecules. These ionic bonds are now widely involved in self-assembly processes leading to the creation of a specific branch in supramolecular chemistry called ionic self-assembly (ISA). Examples of structures made through ISA include ionic self-complementary peptides [50,51] and assembly of surfactants [52,53] and lipids [54] in polyelectrolytes [55].

In contrast to ionic bonds, covalent bonding form between two atoms of similar electronegativities. Such atomic bonds have typical binding energies ranging between 150 and 1100 kJ·mol^−1^ and arise from the equal sharing of one or more electron pairs between atoms in order to achieve an optimum configuration. Generally, this sharing of electrons is not exactly equal, one or more electrons can belong predominantly to one of the atoms involved in the bond, thus forming a polar covalent bond. Therefore, in some cases, it might be difficult to distinguish ionic and covalent bonding as ionic bonds may contain covalent characteristics and reciprocally. Due to their strength and directionality, covalent bonds are not classified as one driving force for the formation of self-assembled systems. However, some aspects of these bonds can induce structural flexibility and reversibility. Recent applications of dynamic covalent chemistry (DCC) highlighted the possibility to easily and reversibly fabricate nanostructured complexes through self-assembly strategies. Accurate control of thermodynamic conditions was found to trigger and control the formation of chemical gradients within self-assembled monolayers [56] and reversible formation of micelles [57]. Isomerization is another example of structural flexibility offered by covalent bonds. Photochromic groups, essentially azobenzene derivatives, have been extensively incorporated into building blocks of supramolecular systems thus allowing the formation of photosensitive materials with switchable architectures [58,59] and properties [60,61].

A special type of covalent bonding is coordination bond. Contrary to conventional covalent bonds where pairs of electrons are equally shared between atoms involved in the bond, in coordination bond all electron pairs are provided by only one of the atoms. Such bonds result from the reaction of a central metal atom or ion reacting with a molecule, atom or ion called ligand. Ligands, also called complexing agents, are Lewis bases able to donate at least one electron pair to a Lewis acid (*i.e.*, central metal atom or ion) through a donor atom. In a coordination complex, the central atom can be bound to one or more ligands thus creating the coordination sphere. Due to the predictable nature and geometries of the metal-ligand coordination sphere, coordination chemistry has been regarded as a powerful tool for the conception of supramolecular complexes. In addition to this predictability, typical energies of coordination bonds (60–300 kJ·mol^−1^) are weaker than usual covalent bond but stronger than hydrogen or aromatic interactions, which make them ideal candidates for the synthesis of flexible and adaptable supramolecular architectures. Recent examples of adaptive and functional structures made through coordination-driven self-assembly include flexible macrocyclic structures [62], molecular flasks [63], sequential self-assembly in metal-organic frameworks (MOFs) [64] and various types of molecular polygons and polyhedra [65,66].

Flexibility and adaptability of supramolecular architectures is a predominant need for applications that require conformational changes. As depicted in this section, all physical and chemical bonds involved in the construction of supramolecular edifices have a certain degree of flexibility regardless of their bonding strength. Following sections are focused on how one can take advantages of the intrinsic flexibility of weak and strong bonds to design functional nanostructured materials. It is worthwhile to note that interactions between building blocks are not the only factors to consider while studying self-assembly. Such processes are thermodynamically driven; therefore, other factors such as enthalpy and entropy can also affect the formation of the final supramolecules and control their physical properties [67,68].

## 3. Flexibility of Organic Nanostructures

Based on the nature of building blocks, self-assembled supramolecules can be classified into organic, organic-inorganic and metal-organic nanostructures. The self-assembly of the first two is mainly driven by weak intermolecular forces, while the last one involves relatively strong coordination bonds between molecular species. The weak intermolecular forces endue the organic nanostructure of a highly dynamic capability or, in other words, these structures can change their conformations with little energy input. A pertinent illustration is the controlled folding of planar graphene nanostructures driven by van der Waals interactions between carbon atoms and water molecules potentially leading to the fabrication of more sophisticated structures such as scrolls, sandwiches, rings and nanosacks [69–71]. Many other examples can be found in living organisms, including the double helices of DNA, the secondary structures of protein, and the lipid bilayers in cell membranes. Highly dynamic changes include the association and dissociation of helices via hydrogen-bonding, folding and unfolding of proteins via amino acid pairing, as well as mass or energy input/output through cell membranes via van der Waals interactions. Inspired by the Nature, people have been able to design and build dynamic nanostructures using synthesized organic molecules. Among them, artificial DNA nanostructures [72–75] and block copolymer thin films [76–78] are relevant representatives, which have gained popularity and fame from early on. In this section, we intended to limit our discussions to these two cases.

### 3.1. Functional DNA Nanostructures

DNA nanotechnology involves the design and manufacture of artificial structures from nucleic acid. Thanks to the folding of oligonucleotide “staple” strands [79], nucleic acids can be assembled into arbitrary 3D structure [80–84]. This programmable assembly is enabled by molecular recognition between complementary strands of DNA, which governs the specific binding of strands with complementary base sequences.

**Figure 2:**
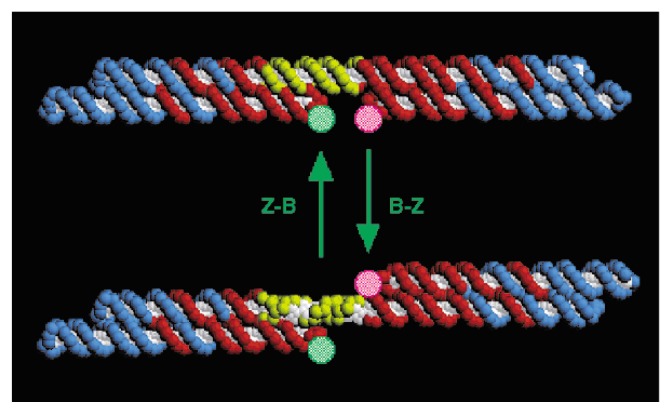
Top, molecular model of the molecule constructed entirely from right-handed B-DNA. Each nucleotide is shown as two spheres, a colored one for the backbone and a white one for the base. Three cyclic strands are shown, one in the center drawn as a red strand with a central yellow segment, and two blue strands on the ends that are each triply catenated to the red strand. Fluorescent dyes are drawn schematically as stippled green (fluorescein) and magenta (Cy3) circles attached to the free hairpins near the middle of the molecule. At the center of the connecting helix is a 20-nucleotide region of proto-Z DNA in the B-DNA conformation, shown in yellow. When the B–Z transition takes place, this same yellow portion becomes left-handed Z-DNA (bottom). When the transition occurs, the two DNA double crossover molecules change their relative positions, increasing the separation of the dyes. The switching event induces atomic displacements of 2~6 nm. Reprinted with permission from [88]. Copyright 1999, Nature Publishing Group.

The flexibility of hydrogen bonds within DNA enabled the construction of artificial DNA nanostructures with the ability to reconfigure upon stimulus, making them one type of nanorobots [85,86]. These artificial structures are constructed as the static structures made from the conventional DNA nanotechnology, but are specifically designed to allow a certain degree of reconfiguration after the initial assembly [85,87]. Such a reconfiguration is usually triggered by interactions with specific molecules or by modification of their environment [88–92]. With this responsive behavior, dynamic DNA nanostructures have found applications in a variety of domains such as molecular sensing, intelligent drug delivery and programmable chemical synthesis [93–96]. Earliest examples of dynamic DNA structures include the use of the twisting motion between the B- and Z-DNA forms to respond when solvent conditions are modified (Figure 2) [88]. This environment-induced transition is able to change the state of all DNA devices in the solution. However, when specific fuel strands are available, multiple devices could perform their motions independently [89,92]. DNA is also used to create opening/closing systems. Which are designed to accurately control the release of a functional cargo under specific conditions [86,96,97]. For example, Douglas *et al.* used DNA origami to develop hollow nanocontainers [96]. The barrel structure consists of two domains covalently fastened in the rear, and non-covalently attached in the front by staples modified with DNA aptamer–based locks [96]. When the aptamer detects the binding key, the nanocontainer is opened and the drug inside the barrel is revealed.

**Figure 3:**
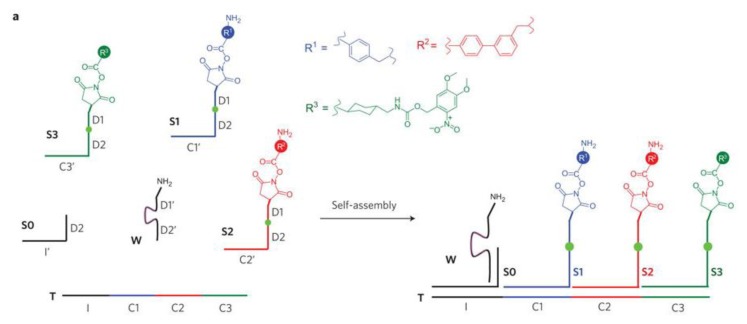

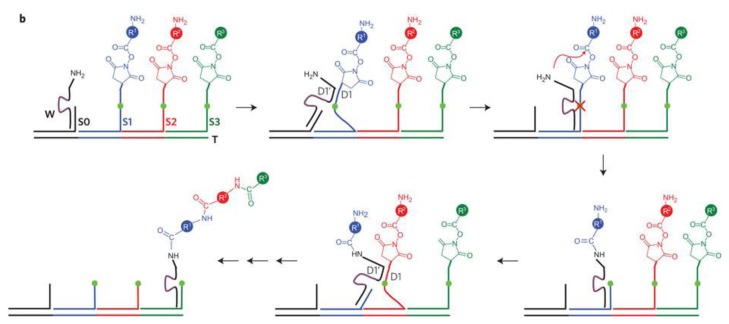
Overview of the DNAsome system. (**a**) The system comprises six DNA or DNA-linked molecules. Three substrates (S1–S3) and an initiator (S0) can hybridize on a single-stranded DNA track (T). Each substrate has an amino acid NHS ester at its 5′ end and two ribonucleotides (green dot) in the middle of its DNA sequence. The DNA walker (W) contains a 3′ amine group and an RNA-cleaving DNAzyme (purple line) that can cleave the ribonucleotides in the substrates; (**b**) DNAsome-mediated multistep synthesis of a triamide product. All steps take place in a single solution under one set of reaction conditions without external intervention. The DNA walker has the ability to pick up and move molecular cargo at each step. Reprinted with permission from [100]. Copyright 2010, Nature Publishing Group.

Hydrogen bonds are not the only interaction able to confer flexibility to DNA. For instance, stronger covalent bonds between nucleic acids of a same strand can be successively cleaved and rebounded. This feature was then used and developed to create DNA walker. DNA walkers are a class of nanomachines that exhibit directional motion along a linear track or on a 2D surface [98,99]. A large amount of strategies have been investigated [85,98]. One of them is to direct the motion along the track using control strands that are manually added in sequence [99,100]. Another way is to utilize enzymes to cleave the strands and cause the walker to move forward, which has the advantage than moving autonomously [101,102]. A later example has been revealed able to walk on a two-dimensional surface rather than a linear track. It also demonstrated the ability to selectively pick up and move molecular cargo at each step (Figure 3) [100,103], making this technique useful in programmable chemical synthesis. Additionally, the walk speed of a DNA walker can be accelerated by using DNA catalysts [104].

All of the above stimuli-responsive behaviors involve multiple DNA strands. Nevertheless, modification of solvent pH was found to trigger the conformational change of a single DNA strand [90,105–107]. For example, a four-stranded short structure can unfold into a double-stranded structure where the length or thickness doubles during the transition. Possibly, such variations of volume and surface roughness could enable future applications in constructing superhydrophilic/superhydrophobic surfaces or molecular motors for MEMS.

### 3.2. Stimuli-Responsive Polymer Systems

**Figure 4:**
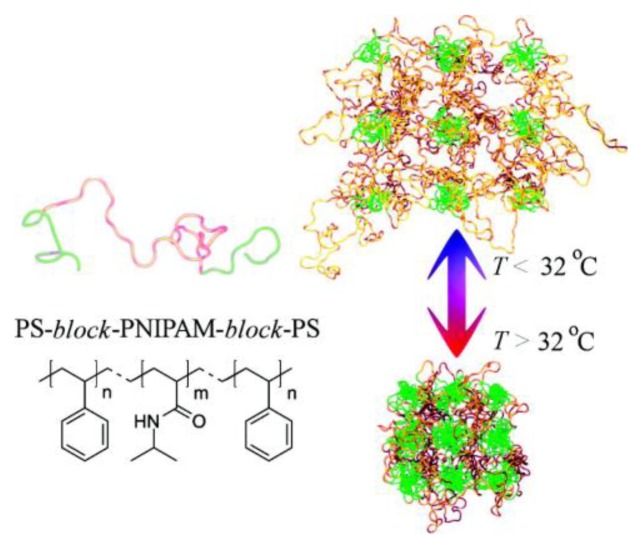
chemical structure of polystyrene-*block*-poly(*N*-isopropylacrylamide)- *block*-polystyrene triblock copolymer. a schematic illustration of temperature-induced conformation transition of aqueous hydrogel having self-assembled morphology with spherical PS domains. The latter domains act as physical cross-links for the hydrogel, and as the temperature is raised above the coil–globule transition temperature the PNIPAM chains become hydrophobic and the gel collapses. Reprinted with permission from [116]. Copyright 2007, American Chemical Society.

Molecular recognition between polymer chains is essential for them to assemble into well-ordered structures. Polymers are composed of repeating units, called monomers, and can be either totally amorphous or semi-crystalline. In the last couple of decades, the assembly of binary or ternary units to form block copolymers has received a close attention particularly in terms of formation process and final structure [108–111]. Such advances allowed block copolymers to be considered as attractive candidates for the fabrication of responsive thin membranes with nearly monodisperse nanopores [112,113]. For instance, properties of triblock copolymers (ABA or ABC) can be modulated by mixing functional terminal blocks with stimuli-sensitive middle blocks thus leading to the formation of phase-segregated structures where functional domains are embedded within a stimuli-sensitive matrix [114–116]. As an example, Nykanen *et al.* reported the formation of temperature-responsive membranes made from polystyrene-*block*-poly(*N*-isopropylacrylamide)-*block*-polystyrene (*i.e.*, PS-*b*-PNIPAM-*b*-PS) [116]. This triblock copolymer containing hydrophobic polystyrene (PS) end blocks and a temperature-responsive PNIPAM midblock can undergo a coil-globule transition as a function of temperature (Figure 4). Permeability measurements revealed that, when thin films of this copolymer are deposited on top of meso/macroporous polyacrylonitrile (PAN) surfaces, the membrane has a switchable on/off permeability. This switching can be controlled by simple variations of the temperature and permeability was found to increase below the coil-globule transition temperature.

When the molecular recognition process between polymer chains is driven by weak hydrogen bonds, several stimuli, such as pH, temperature and even light, can be used to initiate a structural reconfiguration [117–120]. For example, Lee *et al.* fabricated pH-controlled valves using commercially available track-etched polycarbonate membranes [121]. After modification of the membrane pores with PAH/PSS multilayers, pores show the ability to swell and collapse as a function of pH, which makes this system of great interest for pH-triggered separation of small ionic species or to gate the flow of water in microfluidic channels. The similarity in the repeated structures of block copolymers and DNAs has also led to the eventuality of using block copolymers in programmable synthesis where structural flexibility and tunability are essential.

Overall, the versatility, high flexibility and sometimes bio-compatibility of these artificial organic nanostructures make them promising candidates for applications in a wide variety of domains including chemistry, functional biomaterials, sensors and nanodevices.

## 4. Flexibility of Organic-Inorganic Nanolayers

Another important branch in supramolecular chemistry is the fabrication of organic-inorganic hybrid nanolayered structures through self-assembly strategies. Such molecularly engineered nanomaterials are predominantly used to functionalize surfaces and are particularly appreciated for their versatility. The continuous development of available building blocks and optimization of deposition techniques have significantly advanced their designs for specific applications. Moreover, strong interactions between precursors may confer high chemical, thermal and mechanical robustness. Finally, their processing ease makes them readily accessible at low cost and without substantial difficulty. Direct applications of self-assembled organic-inorganic mono- and multilayers extend over a vast range of domains; therefore, this section is intentionally limited to a non-exhaustive list of recent applications, with special emphasis given to the benefits offered by their structural flexibility.

### 4.1. Functional Coatings

**Figure 5:**
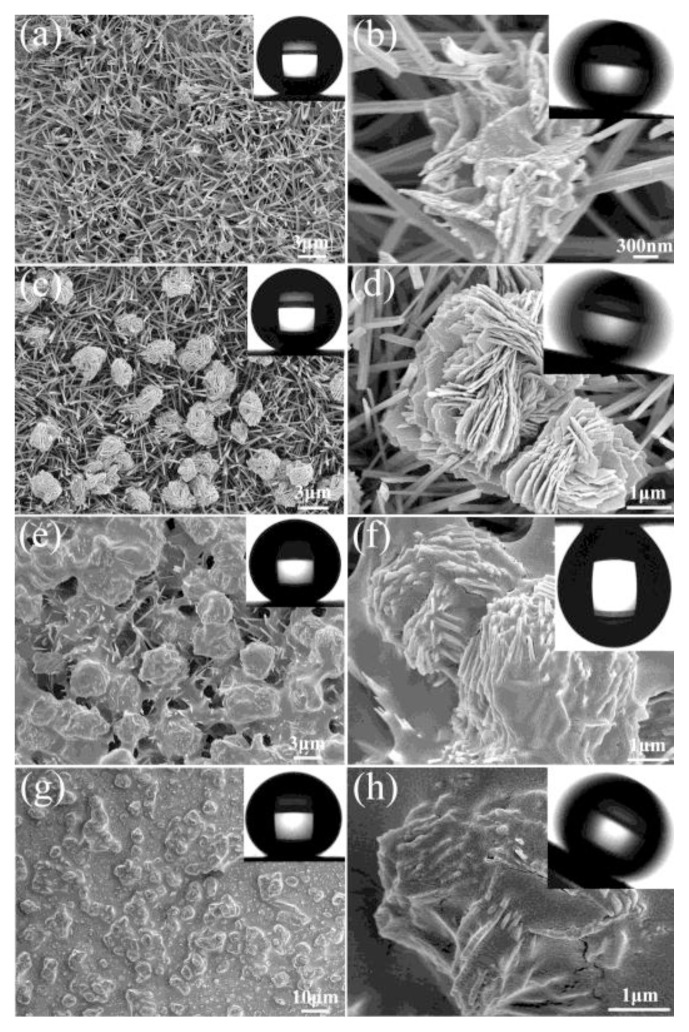
FE-SEM images with different magnifications of CuO films prepared in different reaction times: (**a**,**b**) 5 min, (**c**,**d**) 15 min, (**e**,**f**) 30 min and (**g**,**h**) 60 min. Water droplets on the surfaces shown in the inset. Reprinted with permission from [123]. Copyright 2011, American Chemical Society.

Organic-inorganic nanolayers are widely used as functional coatings to tailor surface properties without altering the integrity of the underlying substrate. Common examples include protective coatings, electronic thin film devices, sensors, robust textiles and biofunctional membranes, where self-assembly and layer-by-layer are the two main strategies for their depositions. For instance, Song *et al.* produced superhydrophobic surfaces by depositing octadecyltrichlorosilane (OTS) self-assembled monolayers (SAMs) on micro/nano-textured silicon substrates [122]. The micro/nano-texture of substrates creates superhydrophobic surfaces with water contact angles (WCAs) of 155° after OTS deposition, as compared to 112° for smooth OTS SAMs. More recently, Li *et al.* reported a convenient and effective method to prepare superhydrophobic surfaces by deposition of fluoroalkylsilane SAMs on CuO surfaces [123]. As shown in Figure 5, tunable water adhesion properties could be achieved by simply controlling topographies of the underlying CuO surfaces through the regulation of the perfluorodecyltriethoxysilane SAM deposition. Highly fluorinated compounds are also used to functionalize polyelectrolyte multilayer films constructed using a layer-by-layer (LbL) deposition technique. For instance, Amigoni *et al.* prepared LbL films by stacking amino- and epoxy-functionalized silica nanoparticles in which the top layer was made hydrophobic by grafting a highly fluorinated monomolecular layer [5]. The hydrophobicity of this hierarchical edifice was found to increase with the number of layers, finally forming stable and highly superhydrophobic surfaces.

**Figure 6:**
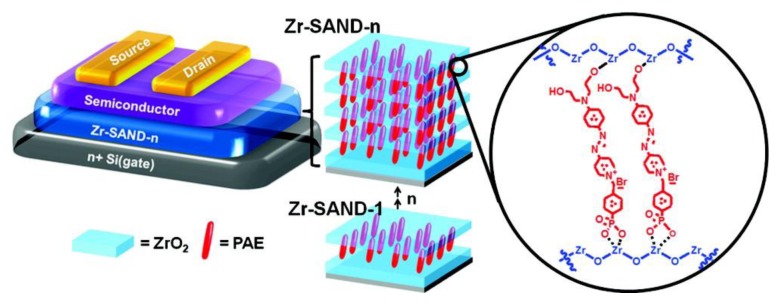
Schematic of a bottom-gate top-contact thin-film transistor (TFT) device geometry incorporating zirconia-phosphonate self-assembled nanodielectric (Zr-SAND). Reprinted with permission from [127]. Copyright 2011, American Chemical Society.

Due to the very large gap between the highest occupied molecular orbital (HOMO) and the lowest unoccupied molecular orbital (LUMO), alkylsilane SAMs can be also used in molecular electronic devices as insulating gates. Ever since the pioneering studies in 1993 [124,125], organic-inorganic mono- and multilayer films gained significant attention for their tunable thicknesses, processing ease and insulating properties leading to their integration in organic transistors [6,7,44,126,127]. Especially, recently developed self-assembled nanodielectrics (SANDs) were found to exhibit promising properties for a variety of opto-electronic applications, including thin-film transistors (TFTs) (Figure 6) [7,126–128]. This new class of gate dielectrics consisting in the stacking of ordered active molecular assemblies onto solid surfaces allows optimizing device performance by their robust insulating properties.

Cohesion and ordering is of paramount importance when fabricated self-assembled structures are used in molecular electronic devices. Poor ordering in molecular packing can lead to reduced performances [129–131]. Therefore, strategies are developed to gain a better control of structural organization. Among them, molecular intercalation has been revealed as a simple and efficient pathway, where ordered nanostructure assembled from a binary system can be easily reconfigured by taking advantage of the reversible bonds during self-assembly [132]. In this approach, addition of foreign molecules to a solution of already formed supramolecular aggregates is used to trigger a major structural alteration. Essentially, the reversibility of weak physical bonds holding tightly packed molecular aggregates allows foreign molecules to be intercalated within the former supramolecules, thus triggering a structural reorganization. Depending on the chemical nature of the building blocks, significant cross-linking or stabilization of the final nanostructures may be introduced after this intercalation process. So far, direct applications of the molecular intercalation method have demonstrated remarkably enhanced thermal [132] and mechanical properties [133] of films after foreign molecules have been accommodated.

### 4.2. Stimuli-Responsive Nanolayers

Switching properties and dynamic functions are highly attractive for construction of sophisticated devices, sensors and “smart” materials. Predictable molecular packing, low energy bonding and ease of processing make self-assembled structures ideal candidates as dynamic and stimuli-responsive materials. These “smart” materials can be activated through several types of external stimulus, such as light exposure, temperature, pH variations, mechanical forces and electromagnetic fields.

**Figure 7:**
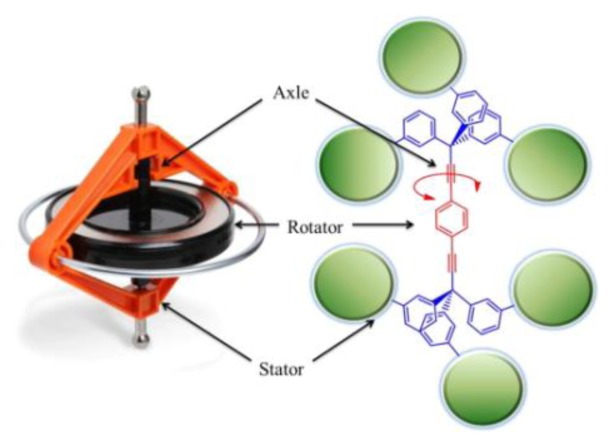
Diagram showing the analogy between macroscopic and molecular gyroscopes. Reprinted with permission from [139]. Copyright 2012, American Chemical Society.

**Figure 8:**
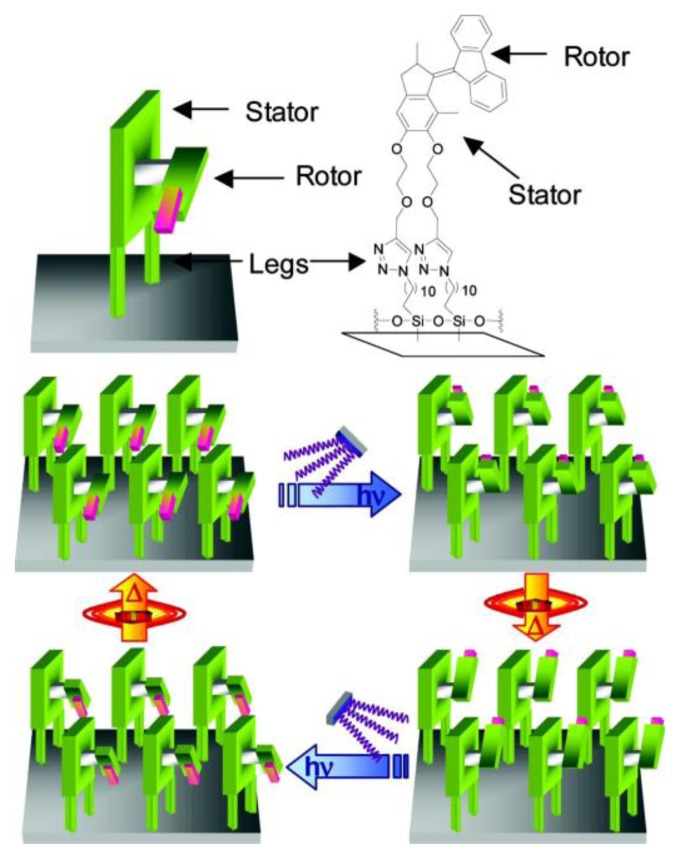
Controlled chemical attachment of light-driven altitudinal molecular motors to a solid substrate provides a monolayer of nanoscale motors. The four stages of the 360° rotary cycle can be addressed with light and heat. Reprinted with permission from [138]. Copyright 2011, American Chemical Society.

Among them, molecular rotors exhibit promising dynamic features that are designed to perform nanomachine-like tasks at the molecular level. Potential applications of these molecular rotors to power artificial nanomachines have already been demonstrated and are incessantly developed [134–136]. They are commonly defined as molecules composed of two parts that can rotate relative to each other. Specifically, they contain a stationary part (the stator) with a large moment of inertia and a part able to rotate (the rotator) with a smaller moment of inertia. Such molecules can form amphidynamic molecular crystals consisting in a rigid lattice composed of axles and bulky static groups as well as mobile parts performing the rotary motion (Figure 7). Recently, Vogelsberg *et al.* investigated the effects of reduced dimensionality on rotational dynamics of p-phenylene moieties confined in organic-inorganic hybrid nanolayers [137]. Examination by variable-temperature NMR revealed a sharp motional change of the rotators with temperature. They postulated that the system undergoes a temperature-triggered transition from a relatively rigid to a fluid-like medium, thus providing an additional insight on the responsiveness and tunability of such materials. Carroll *et al.* also investigated the effect of confinement on rotational dynamics but within a different kind of organic-inorganic system [138]. After depositing an azide-terminated self-assembled monolayer they covalently attached photo-sensitive molecular rotors to the SAM surface leading to the creation of nanoscale machinery on surface (Figure 8). They showed that their molecular rotors are able to undergo photochemical and thermal isomerization even after surface confinement. However, these surface-bound motors have a significantly reduced rate of thermal isomerization due to intermolecular interactions.

The aforementioned isomerization process has been extensively used for the design and construction of different types of stimuli-sensitive supramolecular systems to produce switchable properties. Certainly, azobenzene compounds are the most frequently investigated class of molecules able to undergo photo-isomerization. When irradiated with light tuned to an appropriate wavelength (generally in the UVA region) azobenzene compounds undergo *trans→cis* isomerization. Azobenzene isomerization being reversible the thermodynamically less stable *cis* isomer can relax back to the *trans* form either by illumination with visible light or thermally in the dark. As these two isomers can be switched reversibly by light irradiations, azobenzene motifs are ideal candidates for the construction of photo-sensitive molecular switches and functional materials. While the exact isomerization mechanisms of azobenzene compounds are still under discussion [140], they already found sophisticated applications in a wide variety of domains including biological systems [141], polymers [142], electronics [143], fluidization lithography [144], textiles [145] and drug release [146]. In supramolecular chemistry, the photoswitchable conformation of azobenzene motifs has been extended to control successive self-assembly and disassembly of building blocks [59,147,148], and to reversibly tune functional properties of resulting supramolecules. SAMs of organometallic and organometalloid compounds are the most frequent examples of self-assembled nanolayers containing azobenzene chromophores. Such SAMs are essentially designed for conceiving switches in molecular electronic devices [149–151] and surfaces with controllable properties [152,153]. However, regardless of the applications, one prevalent obstacle has to be overcome. Due to the tight packing of molecules within SAMs, the photoswitching of azobenzene units can be hindered which greatly limits device performances. Several strategies have been pursued to solve this problem. They all aim to laterally space photo-sensitive units from each other to enable a complete and reversible isomerization. Examples of these strategies include co-adsorption of photo-sensitive and inert building blocks [154,155], the use of bulky tripods as anchoring headgroups [156,157], adsorption on porous network [158,159], and addition of lateral group within building blocks to increase the occupied area per molecule [160,161].

**Figure 9:**
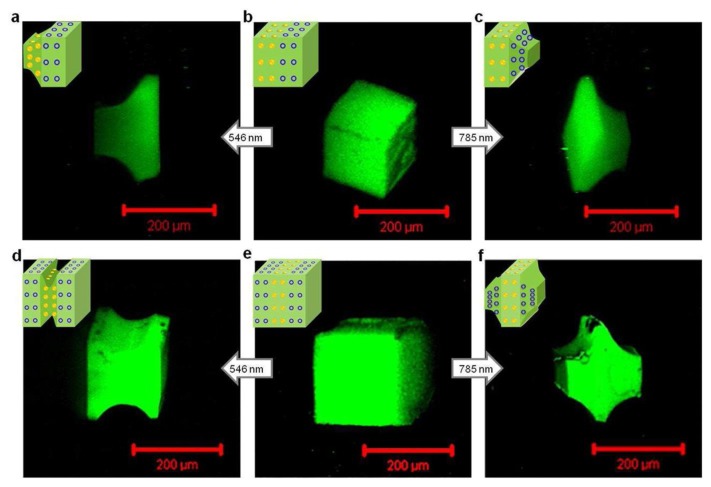
Wavelength-selective shape changes in stratified assemblies of polymer-grafted gold nanoparticles and nanoshells. (**a**–**c**) Confocal laser scanning microscopy images of light-controlled shape changes in two-strata bNP_500_-bNS_500_ cubes upon irradiation using wavelengths at 546 nm (1.1 W/cm^2^) and 785 nm (2 W/cm^2^); (**d**–**f**) Similar experiments with three-strata bNS_300_-bNP_400_-bNS_300_ cubes. Illumination time was 20 min. Reprinted with permission from [169]. Copyright 2012, American Chemical Society.

Among the self-assembly strategies, the layer-by-layer (LbL) deposition method is frequently employed to prepare stimuli-responsive organic-inorganic films. This surface-mediated self-assembly process involves stepwise adsorption of materials of complementary charges. This versatile technique provides a convenient way to control film composition and morphology at the nanoscale and allows the incorporation of an extended variety of functional and responsive components [162,163]. Through the selection of specific building blocks, the LbL method has demonstrated its ability to fabricate responsive coatings. Temperature variations, changes in pH, mechanical stimulations, exposure to light and electromagnetic fields are the most widely explored stimuli in LbL films. Versatility, processing-ease and tunability greatly contributed to the rapid development of an extensive class of stimuli-responsive LbL coatings whose organic-inorganic hybrid architectures are a modest part. To name a few, Schmidt *et al.* prepared electrostatic-based LbL films incorporating inorganic negatively charged Prussian Blue (PB) nanoparticles and organic positively charged gentamicin sulphate (GS), an antibiotic molecule [164]. Electrostatic interactions between these two oppositely charged components ensure the stability and robustness of the layered structure. However, when a sufficient external electrical stimulus is applied, the PB nanoparticles oxidize thus changing their net charge from negative to neutral. Consequently, electrostatic interactions between the PB particles and gentamicin are broken leading the film to dissolve and the gentamicin drug to be released into the solution. Thus, biocompatible organic-inorganic LbL films can be regarded as good candidates for the fabrication of nanocarriers for drug and gene delivery [165]. Wang *et al.* used a chelation-based strategy to tune the release of DNA from a LbL multilayer film [166]. This method relies on another type of interactions, coordination bonding, between the two components (*i.e.*, DNA and zirconium ion, Zr^4+^). Despite the strength of these bonds, they demonstrated the facile and efficient disassembly of films through their immersion in a solution of sodium citrate. Chelators contained in the solution act as substituents. Their high affinity to Zr^4+^ causes the cleavage of coordination bonds between zirconium ions and phosphate groups in the backbone of the DNA chain and the formation of coordination compounds composed of chelators and Zr^4+^. DNA molecules are then released from the LbL film as they no longer interact with zirconium ions. Other examples of stimuli-responsive organic-inorganic LbL multilayer films include temperature, salt and pH-sensitive clay-containing polymer films [167], photo-responsive microcapsules for drug release prepared by a combination of LbL assembly and sol-gel methods [168] and light-controlled swelling of layered polymer/gold nanoparticles composites (Figure 9) [169].

Even though specific, nanolayered organic-inorganic hybrid materials offer a quasi-infinite number of possible combinations between flexible organic compounds and robust inorganic units. Essentially, the main advantage of these hybrid structures relies on the extended range of inter- and intramolecular interactions between components as compared to pristine organic or inorganic materials. Consequently, mixing these two classes of compounds gives access to a large number of possibilities to induce structural flexibility within self-assembled structures, thus enlarging their potential applications.

## 5. Flexibility in Metal-Organic Frameworks

Different from previous 2D structures, metal-organic frameworks (MOFs) can be regarded as coordination-bond driven or assembled nanomaterials with a 3D porous crystalline structure [64,170–174]. Infinite choices of the building units, *i.e.*, metal centers and organic linkers, have rendered this nanomaterial huge construction choices. Their physical and chemistry properties can be further tuned by functionalizing the organic ligands or the metal ions [175–178]. While many metal-organic frameworks are mechanically rigid, some of them are highly flexible in the variation of cell parameters upon an external stimulus [179–183]. As a result, MOFs can find interesting applications in selective gas adsorption/separation or chemical sensing [184–186]. Mainly, this flexibility that enables the volume change is governed by the host-guest interactions within the MOFs. Kitagawa classified these behaviors into three classes: (a) When pillared layers are concerned, interlayer elongation and shortening can be realized by manipulating non-rigid pillars in between [187–189]; (b) when the organic linkers are able to rotate around the metal centers, the change of the guests can induce 3D frameworks expanding or shrinking without changing the topologies [180,190,191]; (c) when frameworks are interpenetrated, insertion of external molecules can introduce a relative motion or sliding of individual network [192–194]. At the time this classification was made, guest changing was the only revealed reason for frameworks flexibility. However, more recently, it was found that the motions within MOFs can also be caused by temperature variation [195] and mechanical pressure [196,197]. Therefore, we also classify the flexibility of MOFs from the origin of the stimuli, namely, (i) gas pressure induced breathing, (ii) solid structure change induced by guest molecule exchange and (iii) temperature or pressure induced volume change.

### 5.1. Gas Pressure Induced Breathing of MOFs

**Figure 10:**
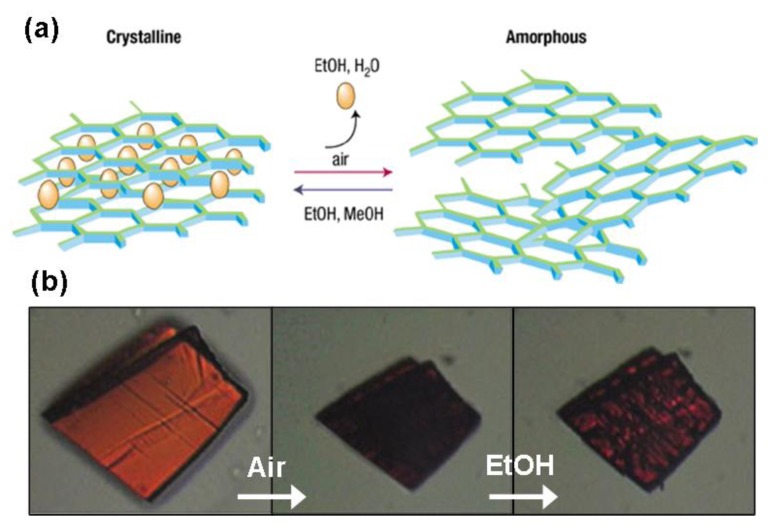
(**a**) The reversible and selective shrinking–breathing behavior of MOROF-1. Reprinted with permission from [189]. Copyright 2003, Nature Publishing Group; (**b**) Images of MOROF-1 crystals followed with an optical microscope. Initially, the crystal was in contact with ethanol solvent. After ethanol removing, the color on the MOROF-1 surface disappeared (middle panel). If a drop of ethanol is placed on the crystal, the lost color appears again (right panel). Reprinted with permission from [188]. Copyright 2003, Nature Publishing Group.

In- or exhale of gas molecules by MOFs is called breathing. This breathing behavior can induce framework movement at a very large scale without destroying the assembly [182]. The largest reversible expansion was found in MIF-88, where the volume change ratio can be as high as 300% [180]. The MIL-88 family is composed of metal carboxylates (chromium or iron) and nitrogen-containing organic bases. Essentially, the knots or vertices inside the framework structures are trimer-like metal clusters that bond with dicarboxylates and form individual bipyramidal cages. A further binding of these cages with the organic base produces one more cavity, *i.e.*, tunnels along [001]. Due to the robustness of the metal clusters and flexibility in binding orientations from the organic bases, distances between the metal clusters at neighboring planes can be changed by simply crumbling or stretching the organic bases without breaking. Typically, the as-synthesized solids can accept solvents with a noticeable and continuous increase in unit cell volume, and when the solvent is taken out, the frameworks shrunk back to the original volume. When this breathing behavior happens in pillared-layered MOFs, such as MOROF-1 [188], along with the volume variation, the crystalline structure could change dramatically (Figure 10). This chemical and structural reversibility of MOROF-1 is accompanied by changes in the magnetic properties, making it a possible candidate for the development of magnetic solvent sensors based on MOFs.

Despite the increasing number of responsive MOFs, further tuning of the dynamic features in frameworks is challenging [183,184,198]. Major focuses were placed on using organic linkers to covalently bond with metal clusters as for [Zn_2_(fu-bdc)_2_(dabco)]*_n_* [183].

### 5.2. Exchange of Guest Molecules

**Figure 11:**
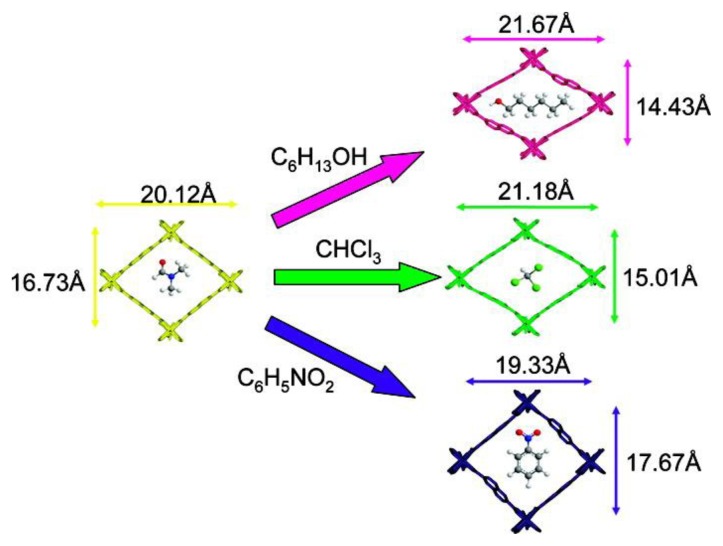
View of the pore systems of individual framework of Zn_2_(NDC)_2_DPNI·DMF (yellow), and the same frameworks with different guest such as C_6_H_13_OH (pink), CHCl_3_ (green) and C_6_H_5_NO_2_ (purple). Reprinted with permission from [194]. Copyright 2009, American Chemical Society.

In addition to the breathing behavior, where the same guest-molecule diffuses into or out of the MOFs, dynamic nature of MOFs is also frequently demonstrated by removal of physically imbedded solvents, followed by a refill with new molecules [194,199]. This exchange of guest molecules can be produced by simple soaking of MOFs in a different solvent. Solvent molecules can diffuse into the porous structure and push the original guest molecules out. For instance, when Zn_2_NDC_2_DPNI·DMF is exposed to 1-hexanol, chloroform, or nitrobenzene, three new structures are formed (*i.e.*, Zn_2_NDC_2_DPNI_2_·C_5_H_13_OH, Zn_2_NDC_2_DPNI_2_·CHCl_3_ and Zn_2_NDC_2_DPNI_2_·C_6_H_5_NO_2_, respectively) [194]. In each case, the new structure shows the complete exchange of the original DMF molecules by the new guest, and is accompanied by significant changes in framework geometry. The most notable change is a lateral movement of atoms along the framework axes defined by the NDC ligands as the frameworks accommodate new guest molecules of different sizes (Figure 11). In order to enable these exchange process, the new molecules should be small enough to diffuse into and settle in the free space of one single cell. Otherwise, the replacement molecules can only replace the guest molecules on the MOFs’ surfaces [200].

### 5.3. External Stimuli

**Figure 12:**
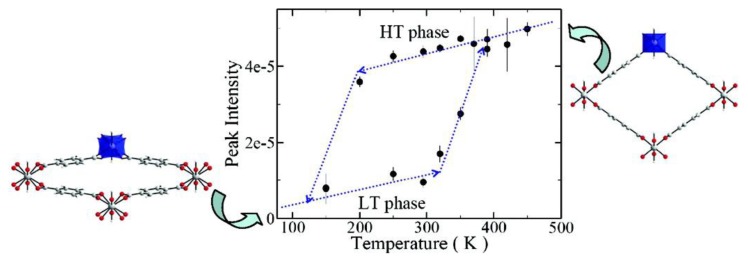
Structural hysteresis diagram of MIL-53(Al) identified using inelastic neutron scattering data measured on the Fermi Chopper Spectrometer. The transition temperature from the high temperature phase to the low temperature phase occurs around 125 K, while reverse transition happens gradually from 325 to 375 K. Reprinted with permission from [195]. Copyright 2008, American Chemical Society.

Both two kinds of movements illustrated above involve exchange with small guest molecules. In fact, movements in an ordered structure can also be performed with an applied field or stimulus, including thermal [195] and pressure variations [196,197]. For instance, the metal-organic framework, MIL-53(Al), is able to close its pores as a function of the applied temperature, without assistance of guest molecules [195]. The observed structural transition shows a significant temperature hysteresis as depicted in Figure 12. During this temperature-triggered pore-opening transition, the longer axis of the structure is slightly elongated from 17 to 21 Å, while the shorter axis shrinks from 13 to 7 Å. Similar transition happens in MIL-53(Cr), when a mechanical pressure is applied [196]. When the external pressure increases from 0.1 to 20 MPa, the longer axis of the structure is elongated from 17.6 to 19.2 Å, while the shorter axis shrinks from 12.2 to 8.6 Å.

## 6. Conclusion

The use of molecular motions offers a promising strategy for the elaboration of self-assembled structures with unprecedented dynamic properties. The outstanding flexibility of weak physical and strong chemical bonds is increasingly employed to develop novel “smart” materials able to perform specific tasks at the molecular level through the application of one or several stimuli.

Remarkably, this strategy can be applied to an extensive variety of material structures, compositions and dimensions. Moreover, as illustrated in this review, numerous properties such as wettability, magnetism, shape, crystalline structure, mechanical robustness and thermal stability, can be easily tuned without substantial effort. This versatility is opening up a wide range of potential applications in multidisciplinary domains including medicine, electronics, sensors, textiles, protective coatings and nanomachinery.

Advances in chemistry are however needed to continue the development of such functional materials. By designing new attractive and responsive building blocks that can be self-assembled, molecular chemistry can continuously broaden the number of available functionalities and their efficiency. However, some intrinsic limitations are still to be overcome in order to gain accurate control of structural modifications and increase material performances.

It is hoped that gaining comprehension of the highly complex interplays existing between building blocks will encourage the development of novel advanced self-assembled materials with the ultimate goal of constructing systems able to accurately mimic dynamic functions of macroscopic machines or even humans.
